# Cell type-specific changes in transcriptomic profiles of endothelial cells, iPSC-derived neurons and astrocytes cultured on microfluidic chips

**DOI:** 10.1038/s41598-021-81933-x

**Published:** 2021-01-26

**Authors:** H. H. T. Middelkamp, A. H. A. Verboven, A. G. De Sá Vivas, C. Schoenmaker, T. M. Klein Gunnewiek, R. Passier, C. A. Albers, P. A. C. ‘t Hoen, N. Nadif Kasri, A. D. van der Meer

**Affiliations:** 1 Applied Stem Cell Technologies, University of Twente, Enschede, The Netherlands; 2 BIOS/Lab on a Chip, University of Twente, Enschede, The Netherlands; 3 Department of Human Genetics, Radboudumc, Nijmegen, The Netherlands; 4 Department of Anatomy and Embryology, Leiden University Medical Centre, Leiden, The Netherlands; 5 Department of Cognitive Neurosciences, Radboudumc, Nijmegen, The Netherlands; 6 Donders Institute for Brain, Cognition and Behaviour, Radboud University, Nijmegen, The Netherlands; 7 Department of Molecular Developmental Biology, Radboud University, Nijmegen, The Netherlands; 8 Centre for Molecular and Biomolecular Informatics, Radboudumc, Radboud Institute for Molecular Life Sciences, 6500 HB Nijmegen, The Netherlands

**Keywords:** Biological techniques, Biotechnology, Cell biology, Computational biology and bioinformatics, Molecular biology, Stem cells

## Abstract

In vitro neuronal models are essential for studying neurological physiology, disease mechanisms and potential treatments. Most in vitro models lack controlled vasculature, despite its necessity in brain physiology and disease. Organ-on-chip models offer microfluidic culture systems with dedicated micro-compartments for neurons and vascular cells. Such multi-cell type organs-on-chips can emulate neurovascular unit (NVU) physiology, however there is a lack of systematic data on how individual cell types are affected by culturing on microfluidic systems versus conventional culture plates. This information can provide perspective on initial findings of studies using organs-on-chip models, and further optimizes these models in terms of cellular maturity and neurovascular physiology. Here, we analysed the transcriptomic profiles of co-cultures of human induced pluripotent stem cell (hiPSC)-derived neurons and rat astrocytes, as well as one-day monocultures of human endothelial cells, cultured on microfluidic chips. For each cell type, large gene expression changes were observed when cultured on microfluidic chips compared to conventional culture plates. Endothelial cells showed decreased cell division, neurons and astrocytes exhibited increased cell adhesion, and neurons showed increased maturity when cultured on a microfluidic chip. Our results demonstrate that culturing NVU cell types on microfluidic chips changes their gene expression profiles, presumably due to distinct surface-to-volume ratios and substrate materials. These findings inform further NVU organ-on-chip model optimization and support their future application in disease studies and drug testing.

## Introduction

Advanced in vitro cellular models are instrumental in understanding CNS function and mechanisms underlying neurological disease. Proper brain function depends on interaction between multiple cell types of the central nervous system (CNS) and its associated vasculature, known as the neurovascular unit (NVU)^1,2^. The NVU is important for cerebral homeostasis and is linked to neurodegenerative diseases^3–5^. Models consisting of multiple cell types are required to study cell type-specific contributions of the NVU to a disorder^6^. Furthermore, it is of interest to study neurological disorders in a patient-specific manner^1^.

Current in vitro neuronal models are typically based on a two-dimensional (2D) cell layer cultured in a microwell plate, which are not designed to controllably mimic the three-dimensional (3D) geometry of nervous tissue. They lack a vascular compartment which prevents their use in studies of neurovascular disease or blood–brain barrier permeability. More complex in vitro neuronal models rely on co-culture of cells in transwell systems, in which cells are cultured on a porous synthetic membrane that is suspended in a well filled with culture medium. Though useful in studying cell–cell interactions, such transwell systems lack key aspects of the geometrical and physical microenvironment of brain tissue. For example, they lack blood vessels with perfusable lumens, and the cell-to-volume ratio of the cell culture medium is non-physiological^7^. Brain organoids are a more complex in vitro neuronal models, consisting of multicellular tissues with a complex 3D geometry than can include vascular structures^8–10^. These models are very useful when studying cell–cell interactions and the CNS microenvironment, however difficulties in controlling their formation results in high variability. They further lack the possibility to provide specific nutrients to individual cell types.

Organs-on-chips are in vitro cell culture models based on microfluidic devices (‘chips’) that integrate human cells, as well as possible sensors and actuators, to simulate the function of tissues or organ subunits^11–14^. The microsystems are designed to offer a specific geometry for the tissue of interest, while also allowing perfusion of liquids and a favourable cell-to-volume ratio. Moreover, the dynamic nature of the system allows for continuous alteration of culture conditions and infusion of solutes at various concentrations. The use of multiple culture compartments gives possibility to culture multiple cell types even when different media are required, and to adjust the fluid shear stress per cell type. Moreover, using patient-specific human induced pluripotent stem cell (hiPSC)-derived cells, these devices can considerably aid the development of personalized medicine^12,15–19^.

The first studies that use organ-on-chips to model the central nervous system and its associated vasculature were published in the past years^20–34^. Increased popularity of these ‘brain-on-chip’, ‘blood brain-barrier-on-chip’ and ‘neurovascular unit (NVU)-on-chip’ models requires a deeper understanding of the biological processes that are influenced by differentiating and culturing cells on microfluidic chips. The geometries and liquid volumes of microfluidic chips differ from those in conventional culture wells, which will likely affect the cells. Previous studies showed the effect of different culture systems, such as 3D culturing, on transcriptomic profiles of cells^35^. Also, co-culturing hiPSC-derived endothelial cells and hiPSC-derived neurons has an effect on their transcriptomes^25^. These studies demonstrate that in vitro cultured cells are sensitive to cues from their culture microenvironment, illustrated by changes in gene expression. Whether culturing on a microfluidic chip induces a gene expression profile associated with a more mature, physiological state of neurons remains to be determined. It is essential to identify culture system-dependent characteristics of various individually cultured cell types when setting up organ-on-chips to model the brain.

In this study we present an open-top microfluidic chip with two compartments that can be used to model the NVU. Cell sources such as iPSC-derived as well as primary cells were used to show the effect of culturing various cell types in different culture environments. The dimensions and materials of a microfluidic chip play a large role in cell behaviour and will likely influence the gene expression profiles of cells. To systematically compare cultures on microfluidic chips and on conventional well plates, we used both these culture systems to study (1) co-cultures of hiPSC-derived neurons (iNeurons) and rat astrocytes, and (2) monocultures of human endothelial cells.

iNeurons were generated by the widely used method of *Neurogenin-2* (*Ngn2*) overexpression, resulting in a homogeneous population of cortical upper-layer II/III excitatory neurons^36,37^. We cultured iNeurons and rat astrocytes together since astrocytes are essential for proper neuronal maturation^38–45^. Human umbilical vein endothelial cells (HUVECs) are a widely used primary cell source in studies of vascular biology; a lot of reference data on gene expression and cell function is available. HUVECs have also been used widely in modelling the NVU and the blood brain barrier in vitro and were therefore used as the primary vascular cell type in this project^29,46,47^.

For each cell type, we determined the changes in gene expression in cells cultured on the microfluidic chip relative to a conventional well plate using RNA sequencing (RNA-seq). Even though the iNeurons and rat astrocytes were cultured together, we could separate the gene expression profiles from both cell types since the cell types originated from different species, enabling us to study each cell type individually. We demonstrate that the implemented culture system affects gene expression in a cell type-specific manner, showing decreased cell division in endothelial cells, increased cell adhesion in iNeurons and astrocytes, and increased maturity of iNeurons when cultured on a microfluidic chip.

## Materials and methods

### Microfluidic chip design

**Figure 1:**
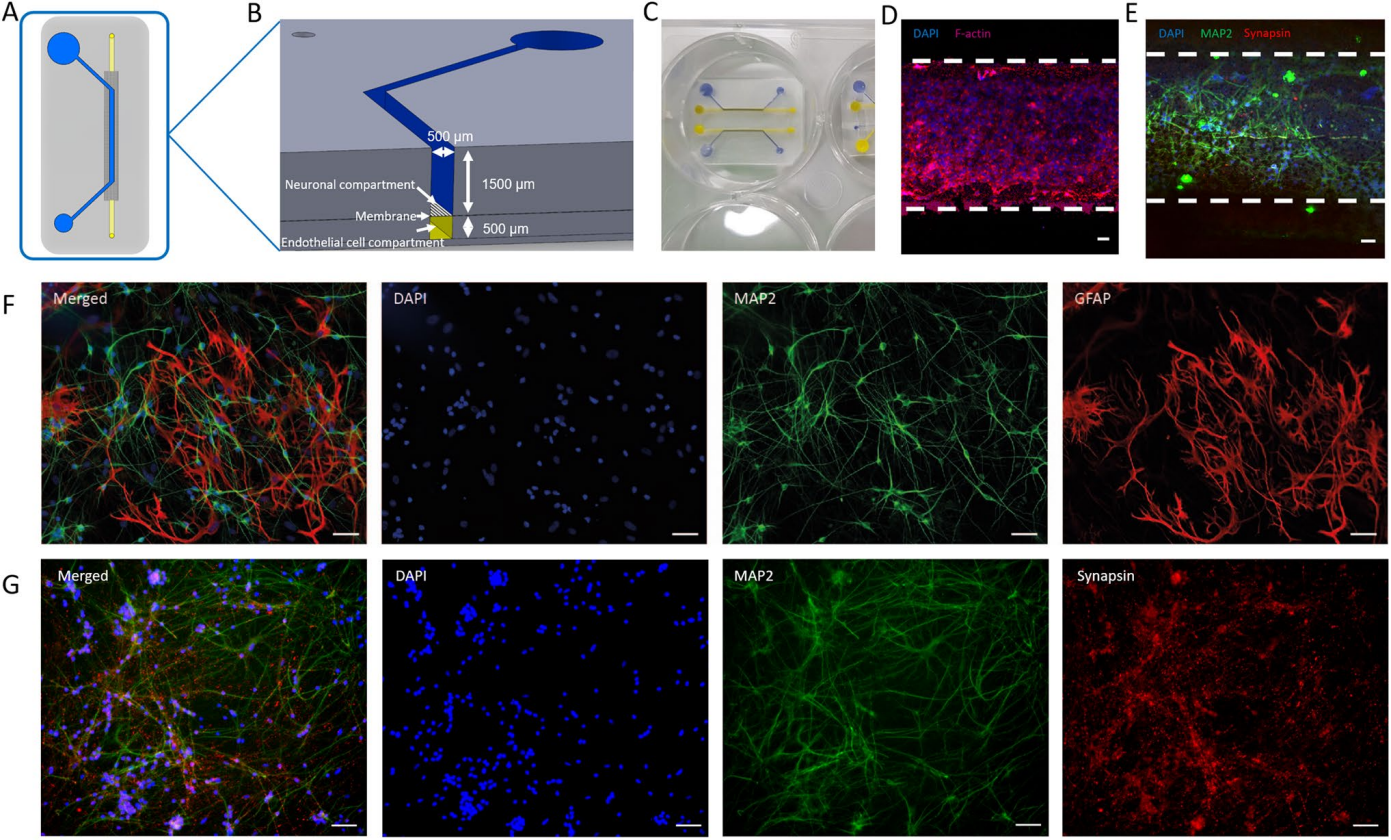
(**A**,**B**) Schematic overview of the microfluidic chip. (**A**) The open top curved channel (blue) is separated from the straight bottom channel (yellow) by a polyester membrane (5 µm pores). (**B**) Cross-section connected channels. (**C**,**D**,**E**) neurovascular unit (NVU)-on-a-chip microfluidic device. (**C**) Open-top microfluidic chip in a 6-well plate, in the bottom channel (yellow) endothelial cells can be cultured, while the open top channel (blue) is used for neuronal differentiation. (**D**) Staining performed on endothelial cell monoculture on bottom channel of the microfluidic chip on a polyester membrane. Based on the staining pattern, a monolayer was present. Blue: Nuclei; Red: F-actin. (**E**) Staining performed on neurons differentiated in the top channel of the microfluidic chip on a polyester membrane. Blue: Nuclei; Green: Microtubule Associated Protein 2 (MAP2); Red: Synapsin-1/2 (SYN1/2). (**F**): Staining performed on neurons co-cultured with astrocytes differentiated on a 24-well plate. Blue: Nuclei; Green: MAP2; Red: Glial fibrillary acidic protein (GFAP), a 1:1 ratio of neurons and astrocytes is visible. (**G**): Staining performed on neurons differentiated on a 24-well plate. Blue: Nuclei; Green: MAP2; Red: SYN1/2. Scale bars, 50 µm.

The microfluidic chip consists of a straight bottom channel (500 µm width × 500 µm height) in which endothelial cells can be seeded, separated from an open-top compartment (500 µm width × 1500 µm height) by a membrane (polyester, 5 μm pore size) (Fig. 1A,B). The design of this chip is similar to other organ-on-chip systems designed to model the central nervous system^20,21,31,32^. The channel dimensions in such chips are optimized to provide a significant co-culture area as well as enough internal volume to culture cells with low flow rates or intermittent medium refreshing. In this study, the open-top compartment was used for differentiation of hiPSCs into iNeurons. The microfluidic chip is designed to fit into a 6-well plate (Fig. 1C), which can be filled with medium while the bottom channel can be perfused separately. The microfluidic chip is designed in such a way that different types of medium can be added to different types of cells (see Fig. 1A,B). The open top allows the treatment of hiPSCs similar to how they would be treated during differentiation to iNeurons or any other cell type in a well plate, while also giving the possibility of adding more volume to the well, therefore decreasing the need for changing medium too often. The bottom channel can be addressed separately by using medium-filled pipet tips, which can be placed on both the inlet and outlet of the channel.

### Microfluidic chip fabrication

The microfluidic chip was fabricated by conventional polydimethylsiloxane (PDMS)-based soft lithography using poly(methyl methacrylate) (PMMA, Arkema innovative chemistry) moulds. Two moulds (one defining the top channels, and one defining the bottom channels) were produced by micromilling (Sherline, model 5410) based on designs in SolidWorks (Dassault Systèmes, France). Polyester porous membranes (Whatman Nuclepore; 5 µm pore size) were cut and positioned on top of the structure that defines the lower channel (Supplementary Fig. 1). Both moulds were pressed together by clamping to prevent leakage and irregularities in the end product. The space between the clamped moulds was injected with PDMS (10:1 base:crosslinker ratio) using a 10 ml syringe. PDMS was left to cure overnight at 65 °C after which it was removed from the mould. The surfaces of both the device and a round 32 mm glass coverslip (menzel-gläser) were activated by exposing them to air plasma (50 W) for 40 s (Cute, Femto Science, South Korea), after which the microfluidic chip was bonded to the coverslip. The activated chip was then coated according to protocol in next chapter.

### Cell culturing of hiPSC-derived neurons and rat astrocyte co-cultures

hiPSC-derived neurons were co-cultured with rat astrocytes, either on a 24-well plate or on the microfluidic chip at 37 °C and 5% CO_2_. For generation and culturing of iNeurons, a previously published protocol^36^ was used that is based on differentiation of hiPSCs (GM25256 iPSC from Fibroblast, Coriell Institute for Medical Research) into neurons (iNeurons) by *Neurogenin 2 (Ngn2)* overexpression using a doxycycline-inducible system. hiPSCs from a commercially available cell line were used, which were transduced with two separate lentiviral vectors containing rtTA and Ngn2^48^. One day before cell plating, channels of the microfluidic chip and wells of a 24-well plate were coated overnight at 4 °C with 20 µg/ml laminin (rhLaminin-521, Gibco) diluted in cold DMEM/F12 medium (Gibco). On days in vitro (DIV) 1 approximately 10,000 cells were seeded per channel and 20,000 cells were seeded per well. To seed cells on the microfluidic chip, the top channels were first filled with culture medium by open-microfluidic flow^49^, after which a droplet containing a high concentration of cell suspension (calculated to contain the correct number of cells) was pipetted into the channels to disperse the cells. The size of the cells prevented them from passing through the membrane to the bottom channel. Chips were incubated for two hours at 37 °C and 5% CO_2_. Two hours after seeding the cells in the channels, the wells containing the microfluidic chips were filled with Essential E8 Flex Basal medium (Gibco), supplemented with 1 × RevitaCell Supplement (Gibco) and 4 µg/ml doxycycline (Sigma Aldrich). For culture in wells plates, the wells were first filled with 500 μl medium, followed by seeding and dispersion of the correct number of cells. From DIV1 onwards, the steps were the same for differentiation of hiPSCs into iNeurons in a well and on the microfluidic chip. On DIV1 cell medium was changed to DMEM/F12 medium, supplemented with 0.1 mg/ml primocin (InvivoGen), 4 µg/ml doxycycline, 1 × N-2 (Gibco), 1 × MEM Non-Essential Amino Acids Solution (NEAA, Gibco), 10 ng/ml NT-3 Recombinant Human Protein (NT-3, Promocell) and 10 ng/ml BDNF Recombinant Human Protein (BDNF, Promocell).

On DIV2, the rat astrocytes (isolated according to earlier described protocols^36^) were added in a 1:1 ratio with the hiPSCs, similar to the hiPSC seeding. A droplet containing a highly concentrated suspension with the calculated number of cells was dispersed on top of the medium. On DIV3, medium was removed and switched to Neurobasal medium (Gibco) supplemented with 0.1 mg/ml primocin, 1× B-27 Supplement, serum free (Gibco), 1X GlutaMAX Supplement (Gibco), 4 µg/ml doxycycline, 10 ng/ml NT-3, 10 ng/ml BDNF and 2 µM Cytosine β-d-arabinofuranoside hydrochloride (Sigma Aldrich). Starting from DIV5, 50% of the medium was refreshed every 2 days with Neurobasal medium, supplemented with 0.1 mg/ml primocin, 1X B-27, 1X GlutaMAX, 4 µg/ml doxycycline, 10 ng/ml NT-3 and 10 ng/ml BDNF. From DIV9 onwards, medium was refreshed every 2 days with this medium, which was then supplemented with 2.5% Fetal Calf Serum (Sigma Aldrich) to sustain the rat astrocytes. iNeurons were kept in culture up to DIV38.

### Cell culturing of human umbilical vein endothelial cells (HUVECs)

Human umbilical vein endothelial cells (HUVECs, Lonza) were cultured either on a 24-well plate or on the microfluidic chip at 37 °C and 5% CO_2_. Cells were seeded to the top surface of a 20 μg/ml laminin (rhLaminin-521, Gibco)-coated bottom channel of the microfluidic chip or to the bottom of a well to reach an initial number of 40,000 cells per channel and 40,000 cells per well. To make sure cells attached to the top of the channel, the microfluidic chips were inverted after cells were added to the channel for at least half an hour. After half an hour, the medium in the channel was replaced with fresh endothelial cell growth medium (ECGM-2: Basal medium (ECBM-2) with supplement mix PromoCell) and microfluidic chips were placed back to the normal upright position. Cells were cultured for 24 h during which medium was changed twice.

### Fixation, staining and imaging of iNeuron and rat astrocyte co-cultures, and endothelial cells

#### iNeuron and astrocyte co-culture

After co-culturing of iNeurons and astrocytes for 30 days on coverslips in a 24-well plate, cells were washed with ice cold phosphate buffered saline (PBS, Gibco). Subsequently, cells were fixated for 15 min at room temperature with 4% formaldehyde (Thermo scientific) and washed three times for 5 min at room temperature with PBS. Cells were permeabilized with 0.2% Triton X-100 (Sigma-Aldrich) solution for 10 min at room temperature. Blocking buffer [PBS (Gibco), 5% normal horse serum, 5% normal goat serum, 5% normal donkey serum, 0.1% bovine serum albumine (BSA), 0.4% triton, 0.1% lysine (all from Sigma-Aldrich)] was incubated for 1 h at room temperature. Primary antibodies (MAP2, Mouse monoclonal, Abcam ab11267, 1:1000; GFAP, Rabbit polyclonal, Abcam ab7260, 1:1000) were diluted in blocking buffer, and were incubated overnight at 4 °C. Cells were washed three times for 5 min with PBS, followed by incubation with secondary antibodies (Goat anti-Mouse IgG Alexa Fluor 488, Thermofisher A-11029, 1:1000; Goat anti-Rabbit IgG Alexa Fluor 647, Thermofisher A-21245, 1:500) diluted in blocking buffer for 1 h at room temperature. Subsequently, cells were washed 3 times for 5 min with PBS, followed by incubation with Hoechst (Thermofisher #H3570) diluted 1:10,000 in PBS for 10 min at room temperature. Lastly, cells were washed one time with PBS for 5 min at room temperature, followed by imbedding of coverslips in fluorescent mounting medium (DAKO #S3023). Cells were imaged using a Zeiss Axio Imager Z1 at a 2752 × 2208 resolution at 20X magnification (scale 1 pixel = 0.23 µm).

#### iNeuron synapsin

After co-culturing of iNeurons and astrocytes for 38 days on chips and well plate, cells were washed with ice cold phosphate buffered saline (PBS, Gibco), fixated for 15 min at room temperature with 4% formaldehyde (Thermo scientific) and subsequently washed 3 times with PBS. Cells were permeabilized with 0.2% Triton X-100 (Sigma-Aldrich) solution for 10 min at room temperature. 5% goat serum (Sigma Aldrich) in PBS was used as a blocking agent for 1 h at room temperature. Primary antibodies (MAP2, Rabbit polyclonal, Abcam ab32454, 1:1000; Synapsin-1/2, Guinea Pig, Synaptic systems 106004, 1:500) were diluted in PBS with 1% goat serum and applied to the cells, which was then incubated overnight at 4 °C. Cells were washed 10 times for 1 min with PBS. Secondary antibodies (Chicken anti-Rabbit IgG Alexa Fluor 488, ThermoFisher A-21441, 1:500; Goat anti-Guinea Pig IgG Alexa Fluor 647, ThermoFisher A-21450, 1:1000) and DAPI (Thermo fisher) were diluted in 1% goat serum and added to the cells. Cells were incubated for 1 h at room temperature. Afterwards cells were washed 10 times for 1 min with PBS. Cells were imaged using a Zeiss LSM 510 confocal microscope at 10X magnification.

#### Endothelial cells

After monoculturing HUVECs for 24 h on a chip, cells were washed with PBS, fixated for 15 min at room temperature with 4% formaldehyde and subsequently washed 3 times with PBS. Cells were permeabilized with 0.1% Triton X-100 solution for 10 min at room temperature. 5% goat serum in PBS was used as a blocking agent for 1 h at room temperature. Alexa Fluor 633 Phalloidin (ThermoFisher A22284, 1:40) and DAPI were diluted in 1% goat serum and added to the cells. Cells were incubated for 1 h at room temperature. Afterwards cells were washed 3 times with PBS. Cells were imaged using a Zeiss LSM 510 confocal microscope at 10× magnification.

### RNA sequencing

**Figure 2:**
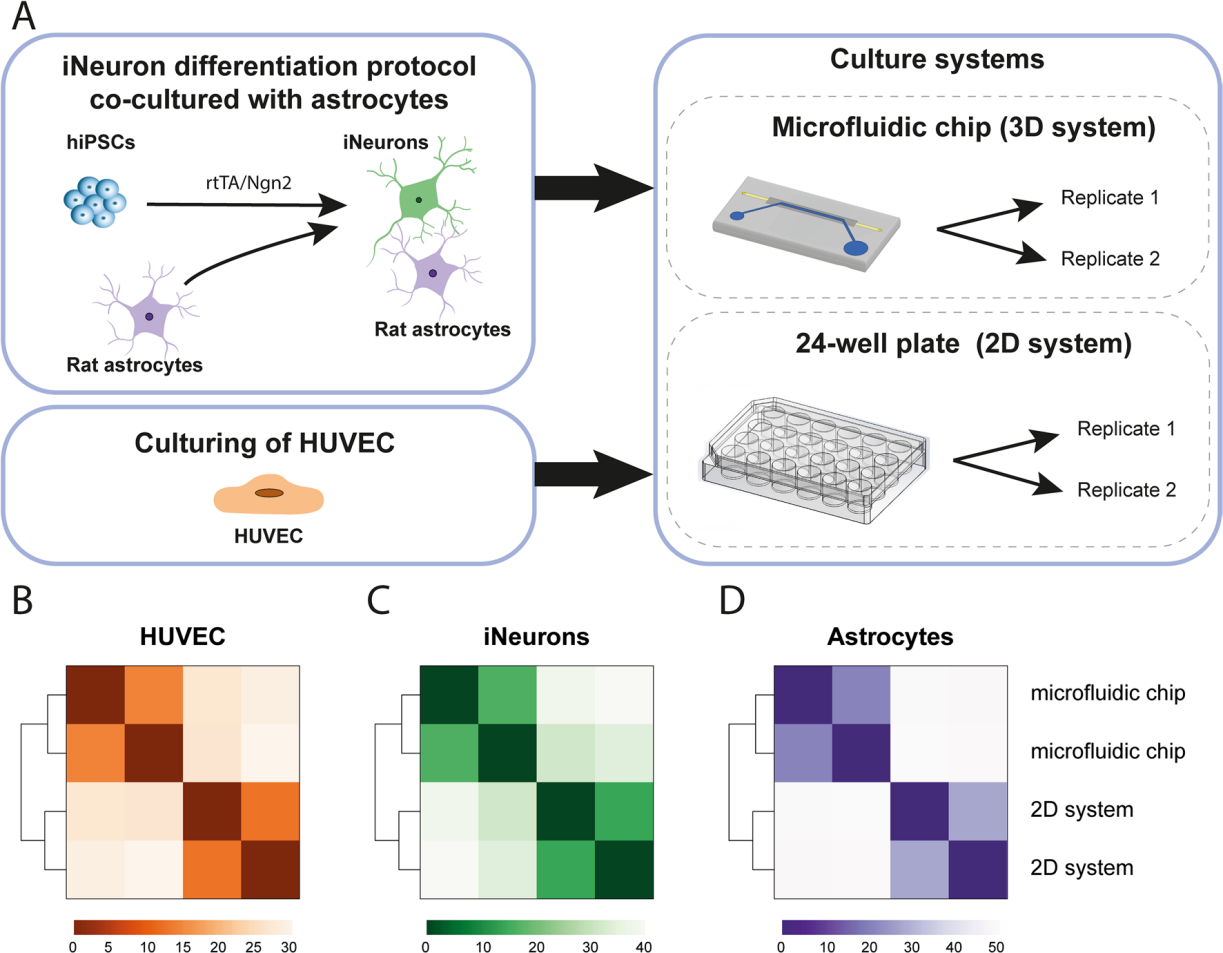
(**A**) Set-up of RNA-seq experiment. hiPSCs transduced with two lentiviral vectors expressing rtTA and Ngn2 are treated with doxycycline to initiate differentiation into iNeurons up to DIV38. On DIV2, rat astrocytes are added to the culture to support neuron differentiation. The experiments were performed both on the microfluidic chip and on a 24-well plate. On DIV38, RNA was isolated from two replicates per culture condition. Separately, HUVECs were cultured on the microfluidic chip and on a 24-well plate as well. After 24 h, RNA was isolated from two replicates per culture condition. (**B**–**D**) Heatmaps of Euclidean distances between samples based on gene expression profiles, generated per cell type (HUVEC, iNeurons, and astrocytes). Within each cell type, samples cluster according to culture condition.

RNA was isolated from monocultured HUVEC samples (24 h after seeding) and from iNeuron and rat astrocyte co-culture samples (DIV38) by adding 100 µl of RNA lysis buffer to the well plate and respectively bottom or top channel of the chip. After the lysis buffer was removed, both plates and chips were checked by using a brightfield microscope for remaining cells and previous step was repeated until no more cells were visible. Cells were cultured either on a microfluidic chip or on a 24-well plate. Two replicates per culture condition were taken (Fig. 2A), resulting in a total of 8 RNA samples. RNA was purified with the *Quick*-RNA Microprep kit (Zymo Research, R1051) according to manufacturer’s instructions. RNA quality was checked using Agilent’s Tapestation system (RNA High Sensitivity Screentape and Reagents, 5067-5579/80). RIN values ranged between 9.0 and 9.8. RNA sequencing (RNA-seq) library preparation was performed using the SMARTer Stranded Total RNA Sample Prep Kit (low input mammalian) (Takara Bio, 634861) according to manufacturer’s instructions. RNA concentrations were determined using the Qubit RNA HS Assay kit (Invitrogen, Q32855). 50 ng of total RNA was depleted for rRNA using the included RiboGone kit (Takara Bio, 634847). After rRNA depletion the remaining amount of RNA was below 10 ng. Library amplification was performed with 12 amplification cycles. Fragment size distribution was determined using Agilent’s Tapestation system (HS D1000 ScreenTape and Reagents, 5067-5584/5). Library concentrations were quantified using the KAPA Library Quantification Kit (KAPA Biosystems, KK4973). Libraries were sequenced on the NextSeq 500 platform (Illumina) using a V2 75 cycle kit (paired-end 2 × 42 bp).

### Pre-processing of RNA-seq data

Base calls were converted to fastq format and demultiplexed using Illumina’s bcl2fastq conversion software (v2.16.0.10) tolerating one mismatch per library barcode. The first three bases of all forward reads were removed using Trimmomatic (version 0.33)^50^, as recommended by the manufacturer of the SMARTer Stranded RNA Sample Prep kit. Trimmed reads from HUVEC samples were mapped to the human reference genome (GRCh38.p12). Trimmed reads from iNeuron samples co-cultured with rat astrocytes were mapped to a combined human (GRCh38.p12) and rat (Rnor 6.0) reference genome, to separate reads belonging to the human iNeurons from reads belonging to the rat astrocytes. Mapping was performed using STAR^51^ (version 2.5.1b) with default settings (–outReadsUnmapped None, –outFilterType Normal, –outFilterScoreMin 0, outFilterMultimapNmax 10, –outFilterMismatchNmax 10, –alignIntronMin 21, –alignIntroMax 0, –alignMatesGapMax 0, –alignSJoverhangMin 5, –alignSJDBoverhangMin 3, –sjdbOverhang 100). Uniquely mapped reads (mapping quality of 255) were extracted. Reads from bam files were further processed to generate count matrices with HTSeq^52^ (version 0.9.1) (parameters: –order = pos, –stranded = yes, –mode = union, –type = exon, –idattr = gene_id). Reference transcriptome GRCh38.p12 (GENCODE 29, Ensembl version 94) was used for bam files from HUVEC samples, and the human reference transcriptome combined with the rat reference transcriptome Rnor 6.0 (Ensembl version 94) was used for bam files from iNeuron samples co-cultured with rat astrocytes. The resulting count tables for the RNA-seq data were used directly for subsequent analyses.

### RNA-seq data analysis

Raw counts from count tables were transformed to counts per million (cpm) using edgeR (R package^53^). Subsequent steps were performed for each cell type individually. Transcripts with a cpm > 2 in at least two samples were included for further analysis (14,069 genes for iNeurons, 12,023 genes for astrocytes, and 12,717 genes for HUVECs). Heatmaps were generated by performing a regularized log transformation on the raw counts using the rld function from DESeq2^54^. Euclidean distances between samples were determined. Differential expression analysis was performed using DESeq2, to compare samples cultured on a microfluidic chip to samples cultured on a well plate. Raw counts were used as input. Genes with a Benjamini-Hochberg (BH)-corrected *p*-value < 0.05 were considered to be significantly differentially expressed (DE) between the two conditions.

Overrepresentation analysis (ORA) of DE genes was performed using goseq^55^. Enrichment of DE genes in Gene Ontology (GO) terms (C5 collection) and canonical pathways (C2 sub-collection CP) from the Molecular Signatures Database (MSigDB, version 7.0)^56^ was determined. Gene symbols corresponding to transcripts that were not included in the DE analysis were removed from the selected gene sets. Subsequently, gene sets with remaining gene set size > 5 and < 500 were used for enrichment analysis. For DE genes identified in rat astrocyte samples, gene symbols had to be converted to its matching human homologue to match with the human gene symbols in MSigDB. Human homologues from the Ensembl homology database (version 94) were used for this conversion, by including one-to-one orthologues and one-to-many orthologues with a confidence score of 1. For 10,653 genes (out of 12,023 included in DE analysis of rat astrocyte samples) human homologues could be identified. For all cell types, human gene symbols (Ensembl version 94) from DE results were converted to gene symbols from Ensembl version 97, corresponding to the version of gene symbols used in MSigDB. DE genes were considered to be significantly overrepresented in MSigDB gene sets for which a Bonferroni-corrected *p*-value < 0.05 was obtained.

For principal component analysis (PCA) count tables from all samples of all cell types were combined. First, counts belonging to rat transcripts had to be assigned to the human homologue of each rat gene. For 16,074 rat genes a unique human homologue could be identified, using one-to-one orthologues and one-to-many orthologues with a confidence score of 1 from the Ensembl homology database (version 94). Raw counts for these 16,074 genes were selected for all samples from HUVEC, iNeurons and rat astrocytes (gene symbols converted to human). The counts were transformed using the variance stabilizing transformation (vst) function from DEseq2. PCA was performed using the prcomp function from stats (R package).

## Results

### Microfluidic chip design and operation

We designed a microfluidic chip and studied the transcriptomic differences of different cell types when cultured on chip versus the traditional well plate method. In our system we can culture cells under similar culturing conditions as when cultured in conventional well plates. This allows us to differentiate hiPSCs into neurons on chip from 0 days in vitro (DIV0) using established protocols. The microfluidic chip has a design similar to other organ-on-chip microdevices that were used in modelling tissues of the central nervous system^20,21,31,32^. The chip contains two channels separated by a porous membrane (Fig. 1A,B). The bottom channel represents the vascular compartment and is used for endothelial cell culture, while the top channel represents the neuronal compartment in which the hiPSC-derived neurons are co-cultured with rat astrocytes. Importantly, the neuronal compartment has an open-top design, which means that it can either be selectively filled with liquids via open microfluidics (Supplementary Movie 1), or it can be exposed to a well fully filled with medium. To seed cells in the top compartment, we used pipetting by open microfluidics, while the long-term culturing was performed by exposing the top compartment to a well filled with medium. The endothelial compartment is a typical microchannel with a closed configuration. We seeded endothelial cells on the porous membrane that forms the top surface of the channel (Fig. 1D). As we directly compare cells cultured on the microfluidic chips and cells cultured in wells, we only evaluated iNeuron and astrocyte co-cultures and HUVEC monocultures in this microfluidic chip. We tested co-cultures of endothelial cells and neurons in the same device as well (see Supplementary section co-culture, Supplementary Fig. 2). However, endothelial cells do not form a full monolayer in those co-cultures, but instead seem to form 3D structures resembling pseudo capillaries. Such structures are often seen when plating endothelial cells on soft, extracellular matrix-like substrates. This may indicate long-term culture of iNeurons leads to changes in the substrate present in the channel of the microfluidic chip on which the endothelial cells are later seeded.

### iNeuron differentiation on microfluidic chip

We generated hiPSC-derived neurons by the widely used method of *Neurogenin-2* (*Ngn2*) overexpression, resulting in a homogeneous population of cortical upper-layer II/III excitatory neurons^36,37^. hiPSCs were differentiated into neurons and co-cultured with rat astrocytes on top of the membrane in the open-top microfluidic chip as well as in a conventional 24-well plate. Because of the different surface-to-volume ratio of the channels on the microfluidic chip compared to wells in a 24-well plate, cell density was adjusted accordingly. The differentiation process was always performed without the presence of endothelial cells. On DIV2, rat astrocytes were added to support differentiation of neurons. We confirmed presence of both iNeurons and astrocytes using immunostaining (Fig. 1F). After 38 days in vitro, cells on the microfluidic chip and in the well plate were fixed and were stained for microtubule-associated protein-2 (MAP2) and synapsin-1/2 (SYN1/2). The images obtained by confocal fluorescence microscopy demonstrate that a network of iNeurons expressing MAP2 and synapsin forms in the top channel of the microfluidic chip, confirming the successful differentiation of hiPSC into neurons on the microfluidic chip (Fig. 1E,G). Interestingly, imaging of the microfluidic co-cultures also showed invasion of neurites from the top compartment through the membrane into the bottom compartment (Supplementary Fig. 2).

### Differences in gene expression patterns between cell types and culture systems

We performed RNA-seq to investigate effects of culturing cells on the microfluidic chip compared to a conventional 2D system. Raw data set have been deposited with the Gene Expression Omnibus (www.ncbi.nlm.nih.gov/geo) under accession code GSE154799^57^. RNA was isolated from HUVEC monocultures (cultured for 24 h), and from iNeuron and rat astrocyte co-cultures (DIV38), cultured both on microfluidic chip and on a conventional well plate. We were interested in the effect of culturing cells on different culture systems for each cell type individually. Since the iNeurons and astrocytes originated from different species, we could separate reads from iNeuron and astrocyte co-culture samples by aligning them to a combined human and rat genome. Gene expression levels were quantified for iNeurons and astrocytes separately by counting reads mapping to human and rat genes, respectively. Gene expression levels for HUVEC samples were determined by aligning reads to the human genome only. As expected, the largest variation between samples on gene expression level was related to cell type differences (Supplementary Fig. 3). Within each cell type, samples clearly cluster according to culture condition (Fig. 2B–D). For further analysis, the effects of culturing cells on a microfluidic chip were studied by comparing samples cultured on the microfluidic chip versus the conventional 2D system for each cell type separately.

### HUVECs cultured on a microfluidic chip show decreased expression of genes related to cell division

Differential expression analysis using DESeq2 was performed on HUVEC samples to identify differentially expressed (DE) genes between the two culture conditions. In HUVEC samples cultured on a microfluidic chip, 853 genes were significantly up-regulated and 1161 genes were significantly down-regulated (adj. *p*-value < 0.05) (Supplementary Table 1). The most significant upregulated genes include *STC1* (logFC = 2.8, adj. *p*-value = 5.64 × 10^–73^), *ITGB4* (logFC = 1.7, adj. *p*-value = 2.77 × 10^–41^) and *ITGA10* (logFC = 1.5, adj. *p*-value = 1.36 × 10^–25^). Interestingly, both STC1 (stanniocalcin-1) and integrins are involved in regulation of tube formation, indicating a more in vivo*-*like growth pattern of the HUVECs^58–61^. The most significantly down-regulated genes included genes important for cell growth and division, including *CENPF* (logFC = − 1.8, adj. *p*-value = 1.76 × 10^–75^), *MKI67* (logFC = − 2.1, adj. *p*-value = 1.76 × 10^–75^), and *TOP2A* (logFC = − 1.7, adj. *p*-value = 3.61 × 10^–61^).

**Figure 3:**
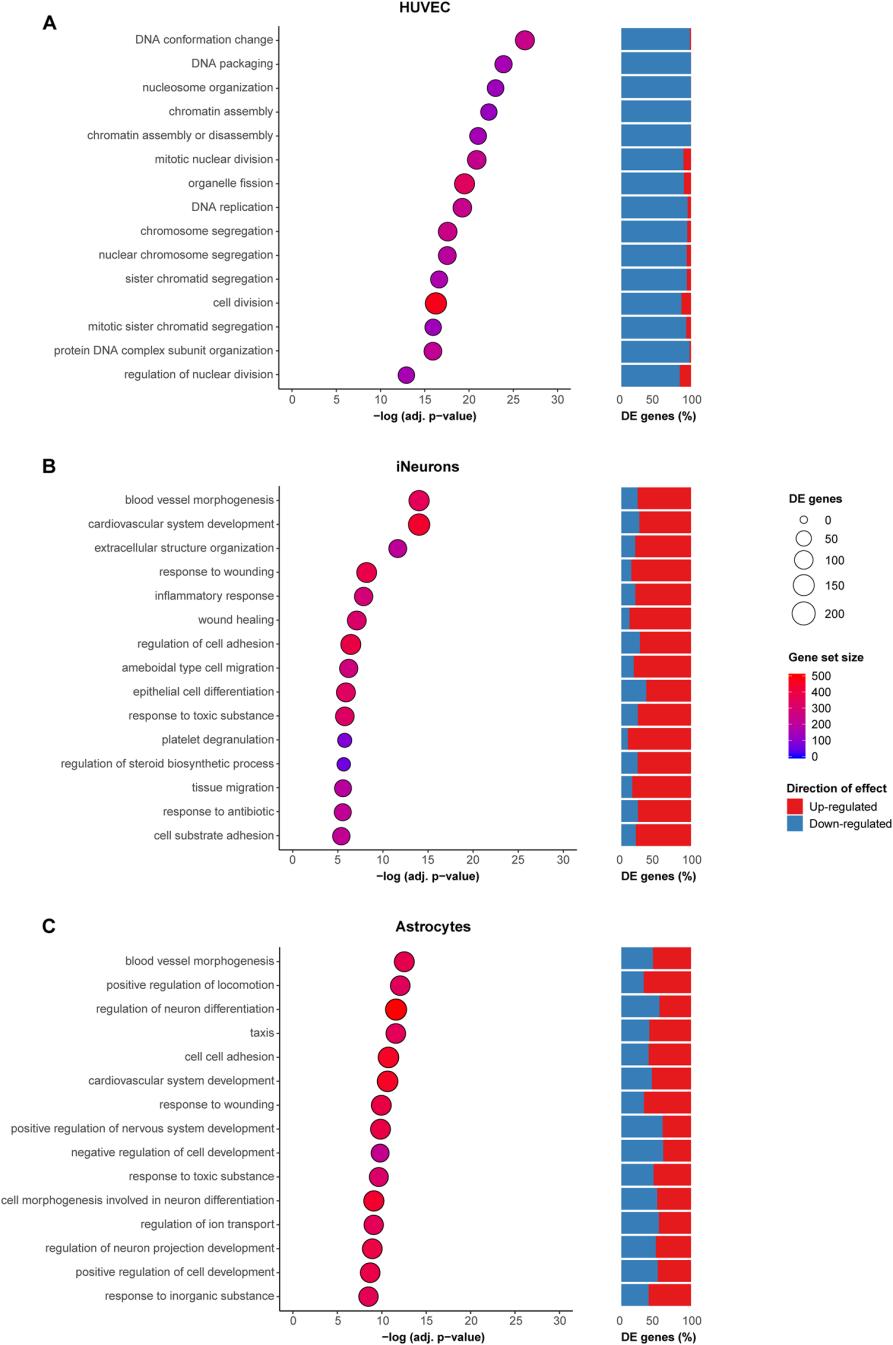
Overrepresentation analysis of DE genes in GO terms was performed by comparing samples cultured on a microfluidic chip compared to a 2D system, for HUVECs (**A**), iNeurons (**B**), and astrocytes (**C**). Top 15 GO terms representing biological processes (BP) are shown per cell type. On the x-axis significance of the overrepresentation is represented by the − log(adj. p-value), calculated by taking the p-value corrected for multiple testing using Bonferroni correction. The gene set size represents the number of genes in each gene set for which a cpm (counts per million) value > 2 was obtained in 2 replicates per cell type, depicted by the color of the circles. The number of DE genes per GO term are depicted by circle size. For each gene set, the percentage of up-regulated (red) and down-regulated (blue) genes of all DE genes per gene set is shown.

Overrepresentation analysis (ORA) showed enrichment of DE genes in 150 gene ontology (GO) terms (adj. *p*-value < 0.05) (Supplementary Table 1). Interestingly, the GO terms were mainly enriched for down-regulated genes. The top GO terms include biological processes (BP) such as regulation of cell cycle, DNA replication and repair mechanisms, and processes involved in cell division (Fig. 3A). The DE genes were also significantly overrepresented in many pathways orchestrating cell cycle (Supplementary Table 1), confirming results from GO terms analysis. Since a high percentage of DE genes in these gene sets was down-regulated, this indicates HUVECs cultured on a microfluidic chip divide and proliferate less. This could be a result of depletion of growth factors in the relatively small culture volume on the microfluidic chip as well as a more in vivo like environment, where endothelial cells are less inclined to proliferate often, when cells are grown on the microfluidic chip.

### iNeurons cultured on a microfluidic chip show increased expression of genes related to cell adhesion, tissue migration and activation of metabolic processes

Differential expression analysis was also performed for iNeurons co-cultured with rat astrocytes. To study gene expression changes in the iNeurons dependent on culture system, we selected reads from the co-culture samples that mapped to the human genome. In total, 1480 genes were significantly up-regulated and 1139 genes were significantly down-regulated (adj. *p*-value < 0.05) in iNeurons cultured on the microfluidic chip (Supplementary Table 2). Interestingly, among the top up-regulated genes were several genes involved in cell adhesion, including *FN1* (logFC = 7.5, adj. *p*-value = 1.08 × 10^–118^), *THBS1* (logFC = 9.1, adj. p-value = 2.31 × 10^–58^) and *MSN* (logFC = 5.4, adj. p-value = 3.47 × 10^–48^). Overrepresentation analysis revealed significant overrepresentation of DE genes in 133 GO terms (adj. *p*-value < 0.05) (Supplementary Table 2). Indeed, these results show overrepresentation of DE genes in cell adhesion gene sets (Fig. 3B). Other GO terms enriched for DE genes represented processes such as extracellular matrix (ECM) organization, tissue migration, and steroid metabolic processes. The majority of these genes were up-regulated, indicating increased activation of these processes in iNeurons cultured on a microfluidic chip. Pathway analysis confirmed the results from GO term analysis (Supplementary Table 2).

Among the top down-regulated DE genes were many transcriptional regulators important for development or involved in regulating cell growth, such as *BHLHE41* (logFC = − 3.2, adj. *p*-value = 4.61 × 10^–44^), *JUN* (logFC = − 1.3, adj. *p-*value = 2.90 × 10^–23^), *TSHZ2* (logFC = − 1.9, adj. *p*-value = 6.78 × 10^–23^), *KLF10* (logFC = − 1.9, adj. *p*-value = 2.86 × 10^–21^), and *RARB* (logFC = − 3.8, adj. *p*-value = 2.98 × 10^–19^). This indicated the process of neuronal differentiation might depend on culture system. Indeed, several neuronal maturation marker genes were significantly differentially expressed in the iNeurons. *SOX1* (logFC = − 2.4, adj. *p*-value = 6.72 × 10^–3^) and *DCX* (logFC = − 0.3, adj. *p*-value = 2.45 × 10^–3^), both neural progenitor (NPC) markers, and *EFNA5* (logFC = − 1.3, adj. *p*-value = 5.18 × 10^–12^), an immature neuronal marker, were significantly down-regulated in iNeurons cultured on the microfluidic chip. Conversely, post-mitotic neural marker *SYP* (logFC = 0.5, adj. *p*-value = 1.89 × 10^–4^), and synaptic markers *SLC17A6* (logFC = 0.4, adj. *p*-value = 2.45 × 10^–3^), *SNAP25* (logFC = 1.1, adj. *p*-value = 7.78 × 10^–16^)*, SYT1* (logFC = 0.3, adj. *p*-value = 0.03), and *SYT2* (logFC = 1.6, adj. *p*-value = 7.04 × 10^–23^) were up-regulated. These results indicate that the iNeurons cultured on a microfluidic chip are more mature compared to iNeurons cultured on the 2D well system.

### Astrocytes cultured on a microfluidic chip exhibit differences in gene expression patterns that regulate neuronal differentiation

Finally, to compare the effect of the different culture systems on rat astrocytes, we selected reads from the iNeuron and astrocyte co-culture samples that mapped to the rat genome. DESeq2 analysis identified 768 significantly up-regulated genes and 752 significantly down-regulated genes from astrocyte samples cultured on the microfluidic chip compared to a conventional well plate (adj. *p*-value < 0.05) (Supplementary Table 3). Interestingly, the most significantly up-regulated gene is *Scg2* (logFC = 5.3, adj. *p*-value = 4.38 × 10^–234^). Scg2 (secretogranin II) can be secreted by astrocytes^62,63^ and is known to regulate neuronal differentiation, providing more evidence for a more mature state of the co-cultured iNeurons on a microfluidic chip. Many other genes relevant for astrocyte-neuron communication were among the most significantly DE genes as well, including *Slc7a11* (glutamate release) (logFC = 4.0, adj. *p*-value = 5.35 × 10^–125^), *Cp* (iron metabolism) (logFC = − 3.2, adj. *p*-value = 5.26 × 10^–76^), *Hgf* (neurotrophic growth factor) (logFC = − 4.0, adj. *p*-value = 1.02 × 10^–35^), and *Atp1a2* (potassium clearance) (logFC = − 2.2, adj. *p*-value = 2.94 × 10^–34^).

We determined whether there is overrepresentation of astrocyte DE genes in GO terms and pathways (Supplementary Table 3). For 10,653 genes (out of 12,023 included in DE analysis) human homologues could be identified, which were used for overrepresentation analysis in gene sets. In total 1411 of these genes were significantly differentially expressed, which were overrepresented in 213 GO terms (adj. *p*-value < 0.05). We investigated whether there was overlap between GO terms identified for the different cell types, and observed large overlap between GO terms identified for iNeurons and astrocytes, whereas there was hardly any overlap with GO terms identified for HUVECs (Supplementary Fig. 4A). The overlapping GO terms between iNeurons and astrocytes include processes such as cell adhesion and tissue migration (Fig. 3C). Interestingly, the overlap in DE genes between iNeurons and astrocytes is not higher than the overlap with HUVECs and either cell types (Supplementary Fig. 4B). This indicates that although the same processes are changed in both iNeurons and astrocytes, these changes are caused by altered expression of different genes. GO terms that were unique for astrocytes mainly represent DE genes that have a function in regulation of cell development and neuron differentiation. Pathway analysis confirmed findings from GO term analysis, with changes observed in pathways such as ECM organization, cell adhesion, axon guidance and regulation of the neuronal system (Supplementary Table 3).

Overall, the results suggest the degree to which astrocytes support neuronal differentiation differs depending on the culture system used. This was shown by the highly significant differential expression of genes relevant for communication between astrocytes and neurons, as well as the significant enrichment of DE genes in GO terms that represent regulation of neuronal differentiation. The function of astrocytes and neurons are known to be closely connected. Astrocytes respond to neurotransmitters released by neurons, whereas neurons respond to factors released by astrocytes that influence synaptic activity and function of neurons^64^. In iNeurons we observed increased expression of marker genes for mature neurons, and decreased expression of marker genes for immature neurons. These findings indicate that iNeurons mature faster when cultured on a microfluidic chip compared to conventional 24-well plates. The changes in gene expression observed in the co-cultured astrocytes support these findings, as they seem to contribute to the difference in neuronal maturation. It could also be that changes in gene expression observed in astrocytes are a secondary result caused by the difference in maturity of iNeurons, rather than underlying the changes in neuronal maturation. Nevertheless, our results show that iNeurons co-cultured with astrocytes display a different, more mature state, when cultured on a microfluidic chip.

## Discussion

In this study we present a new, open-top microfluidic chip for culturing multiple cell types, which we used to study transcriptomic differences of different cell types when cultured on chip versus the traditional well plate method. The chip can be used for future studies to study interaction between cell types as well. We cultured different cell types on these microfluidic chips relevant for modelling the NVU, consisting of (1) co-cultures of hiPSC-derived neurons and rat astrocytes, and (2) monocultures of HUVECs. We were interested to identify processes that are changed in each cell type when cultured on a microfluidic chip, compared to conventional culture systems. These cell type-specific changes in gene expression were investigated using RNA-seq. HUVECs were cultured separately from hiPSC-derived neurons co-cultured with rat astrocytes, both on the microfluidic chip and on a conventional 24-well plate. We demonstrate that culturing cells on microfluidic chips has a clear, cell type-specific effect on gene expression.

Endothelial cells exhibited decreased expression of genes related to cell division and increased expression of genes related to tube formation when cultured on microfluidic chips. The medium used for culturing endothelial cells stimulates endothelial proliferation by high levels of growth factors like vascular endothelial growth factor (VEGF) and fibroblast growth factor (bFGF). The microfluidic chip has a surface-to-volume ratio that is approximately five times higher than that found in the well plate (0.01 cm^2^/µl vs. 0.0019 cm^2^/µl respectively). Therefore, the decrease in expression of genes related to proliferation and increased expression of genes related to tube formation may be due to depletion of growth factors in the relatively small culture volume on the microfluidic chip. Under normal conditions, endothelial cells do not proliferate continuously when in contact with other endothelial cells. Thus, the decreased expression of genes related to cell division can be representative of a more in vivo like environment when cells are grown on the microfluidic chip. These findings stress the importance of adjusting physiological flow accordingly when setting up an organ-on-chip model. Fluid flow will continuously refresh the medium in the bottom compartment to provide endothelial cells with sufficient nutrients but can also cause shear stress; the mechanical stimulus caused by flowing liquid. Previous research has shown different gene expression patterns in HUVECs cultured in a microfluidic chip under different continuous flow profiles^65^. It is important to set the fluid flow at a level where it provides sufficient medium for cell survival, while keeping it at a minimum to prevent shear stress and maintain the cells at a less proliferative state comparable to the in vivo situation.

Gene expression data from co-cultured iNeurons and rat astrocytes indicated iNeurons mature more rapidly when cultured on microfluidic chips compared to conventional well plates. This was shown by increased expression of marker genes for mature neurons and decreased expression of marker genes for immature neurons. It was further supported by findings from the co-cultured astrocytes, showing expression of astrocyte genes involved in regulation of neuron differentiation was affected. The function of astrocytes and neurons are closely connected. Astrocytes respond to neurotransmitters released by neurons, and vice versa neurons respond to factors released by astrocytes that influence synaptic activity and function of neurons^64^. This could explain the changes we find on gene expression in both neurons and astrocytes that are linked to a different neuronal maturation state of neurons.

Furthermore, both iNeurons and astrocytes exhibited increased expression of genes related to adhesion, migration, and ECM organization upon culture on the microfluidic chips. Previous studies have shown upregulation of cell motility, axon guidance and cell morphogenesis^35^ in neurons when cultured in 3D versus 2D, in line with our findings. It is unclear which aspect of the microenvironment accounts for changes in cell adhesion and ECM organization. Both the substrate (PDMS and polyester membranes) and the geometry on the microfluidic chip are different from the flat tissue-culture treated polystyrene found in a well plate and could contribute to these factors.

We show that differentiation of hiPSCs to neurons is possible from DIV0, preventing the need to transfer neurons at a later stage during development. Using this approach, we performed, for the first time, the full differentiation process on the microfluidic chip, from hiPSC to neurons up to 38 days in vitro*.* We also show that co-culture of all three cell types together on a microfluidic chip to obtain functional iNeurons and a separate monolayer of endothelial cells is not possible if the endothelial cells are added after long-term culture of iNeurons. The incomplete endothelial monolayer formation is most likely the result of neurites extending into the endothelial compartment, thereby affecting the surface on which endothelial cells need to attach. Sances et al.^25^ observed the same problem when co-culturing hiPSC-derived spinal cord neurons and endothelial cells and demonstrate the problem can be solved by changing the order of seeding cell types, allowing the formation of an endothelial monolayer before introducing neurons. The use of HUVECs as the primary vascular cell type in our study poses a limitation. Although HUVECs are widely used in modelling the NVU and the BBB in vitro^29,46,47^, other cell types (e.g. primary human brain microvascular endothelial cells) may function differently when cultured with neurons in a microfluidic chip. For example, the incomplete formation of an endothelial layer that we observed upon co-culture could also be the result of not using a different brain specific cell source such as human brain microvascular endothelial cells or even induced brain microvascular endothelial cells^66^. Studies that use such cell types for co-culture with neurons in microfluidic chips have demonstrated clear monolayer formation^67^. Using iPSC-derived cells would also enable a more patient-specific model and prevent potential issues in transcriptomic profile comparisons due to different cell sources.

Altogether, our data show clear cell type-specific responses in gene expression dependent on culture system. We show endothelial cells cultured on microfluidic chip more closely resemble in vivo conditions, and neurons cultured on microfluidic chip are more mature. These results show the microfluidic chip could be used as a useful tool to model the NVU. Our findings can serve as a reference point for future studies towards application of microfluidic chips in modelling the NVU to study its function and its role in pathology of neurological disorders.

## Conclusion and outlook

In this paper we show a new, open-top microfluidic chip that allows for controlled integration of multiple cell types to model the neurovascular unit (NVU). We demonstrate that for different cell types (co-cultures of hiPSC-derived neurons and astrocytes, and monocultures of endothelial cells) separately cultured on the microfluidic chip, cell type-specific gene expression profiles are highly dependent on culture system. These findings can serve as a reference point for future studies to study interactions between cell types, to increase understanding on neurovascular physiology and neurovascular disease. The use of hiPSC-derived neurons from individual patients will give insight in patient variability in disease and allows identification of patient-specific responses to treatment.

## Supplementary Information

Supplementary Video 1.

Supplementary Figures.

Supplementary Table 1.

Supplementary Table 2.

Supplementary Table 3.
