# Supplementary material for: Cell type-specific changes in transcriptomic profiles of endothelial cells, iPSC-derived neurons and astrocytes cultured on microfluidic chips
Source: Sci Rep. 2021 Jan 26;11:2281. doi: 10.1038/s41598-021-81933-x (PMC7838281; doi:10.1038/s41598-021-81933-x)
Supplement: Supplementary file 2 — Supplementary Figures. [file 41598_2021_81933_MOESM2_ESM.pdf]

Cell type-specific changes in transcriptomic profiles of endothelial cells, iPSC-derived neurons and astrocytes cultured on microfluidic chips

H.H.T. Middelkamp[ǂ*1,2]; A.H.A. Verboven[ǂ*3,6,8]; A.G. De Sá Vivas[1,2] ; C. Schoenmaker[3]; T. M. Klein Gunnewiek[3,6]; R. Passier[1,4]; C.A. Albers,[3,6,7]; P.A.C. 't Hoen[8]; N. Nadif Kasri[3,5,6]; A.D. van der Meer*[1]

[1]Applied Stem Cell Technologies, University of Twente, Enschede, The Netherlands

[2]BIOS/Lab on a Chip, University of Twente, Enschede, The Netherlands

[3]Department of Human Genetics, Radboudumc, Nijmegen, The Netherlands

[4]Department of Anatomy and Embryology, Leiden University Medical Centre, Leiden, The Netherlands

[5]Department of Cognitive Neurosciences, Radboudumc, Nijmegen, The Netherlands

[6]Donders Institute for Brain, Cognition and Behaviour, Radboud University, Nijmegen, The Netherlands

[7]Department of Molecular Developmental Biology, Radboud University, Nijmegen, The Netherlands

[8]Centre for Molecular and Biomolecular Informatics, Radboudumc, Radboud Institute for Molecular Life Sciences, 6500 HB Nijmegen, the Netherlands

Supplementary information

Supplementary information

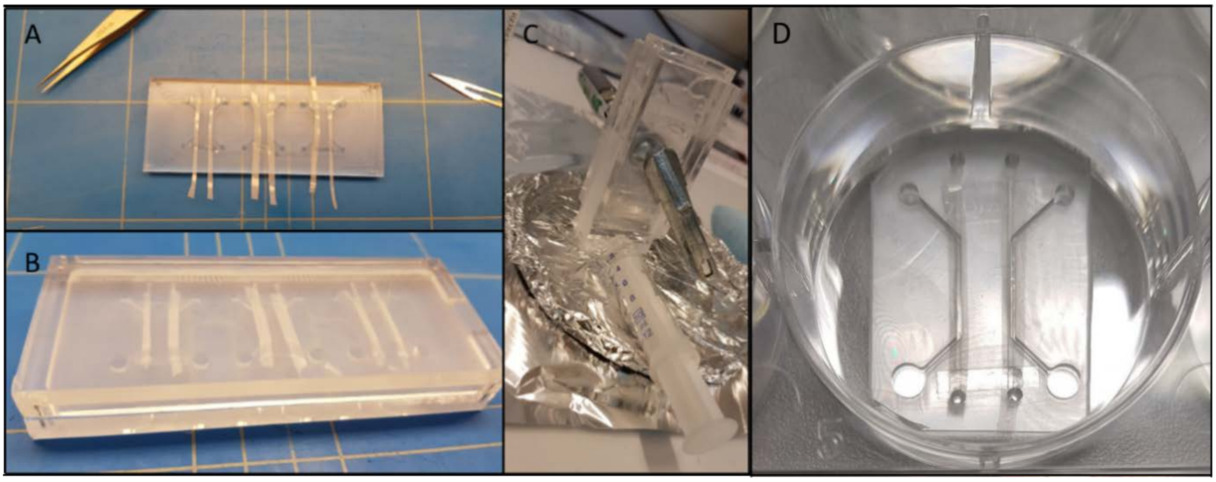

Supplementary figure 1: The process of incorporating the membrane of the microfluidic chip using injection moulding. A: Membranes are cut into slices slightly wider than channel. Using double sided scotch tape, the membranes are attached on top of the curved channels. B: Both sides of the mould (curved and straight channel separated by the membrane are pieced together. C: Using a 12 ml syringe needle approximately 8 ml of PDMS is pushed between the moulds and cured in an oven. D: Microfluidic chips are bonded to a round 32mm coverslip using plasma activation. Microfluidic chips fit into a 6 well plate for convenience.

Supplementary section Co-culture:

Methods:

Culturing of iNeurons and endothelial cells together on the same microfluidic chip was performed similar to culturing of the cell types separately on a microfluidic chip (See methods; section Cell culturing). The bottom channels were coated alongside the top channels with 20 µg/ml laminin on DIV 0. On DIV1 hiPSCs were plated and differentiated into iNeurons on the top channel, with addition of rat astrocytes on DIV2. On DIV37, endothelial cells were seeded onto the bottom of the membrane and co-cultured for 24 hours with the iNeurons.

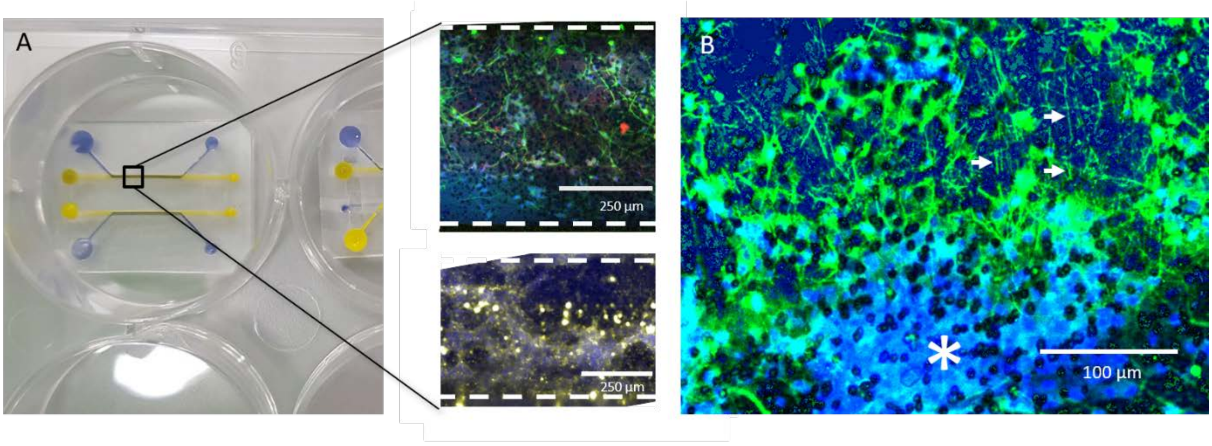

Supplementary figure 2: (Co-)cultures of iNeurons and endothelial cells. A: Staining performed on neurons differentiated in the top channel and endothelial cells in the bottom channel of the microfluidic chip on a polyester membrane. Endothelial cells no longer formed a monolayer, but formed structures resembling typical pseudo capillaries. Blue: Nuclei; Green: Microtubule Associated Protein 2 (MAP2); Red: Synapsin-1/2 (SYN1/2); Yellow: F-actin. B: Neurite infiltration (arrows) through membrane in endothelial (asterisk) field of focus, Blue: Nuclei; Green: MAP2.

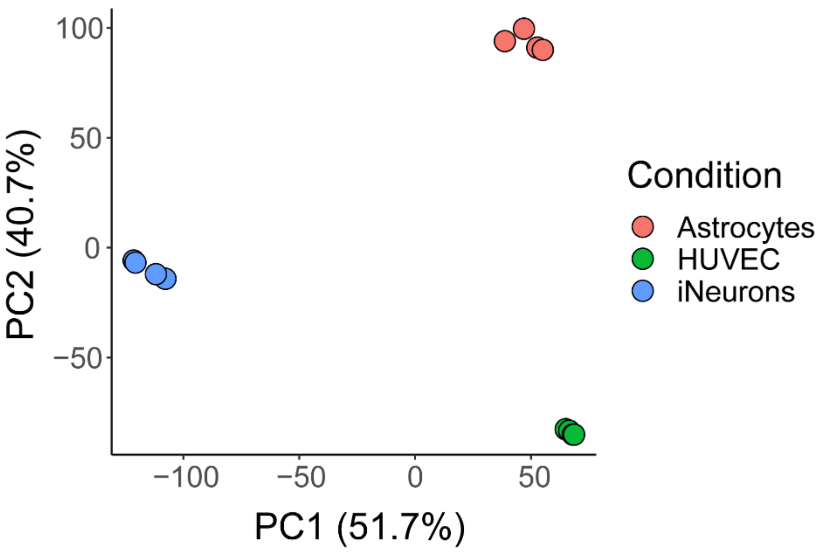

Supplementary figure 3: Principal component analysis (PCA) performed on HUVEC, iNeuron and astrocyte samples, including all genes for which a human homologue is available (n=16,074). PC1 and PC2 are shown. Samples are colored per cell type.

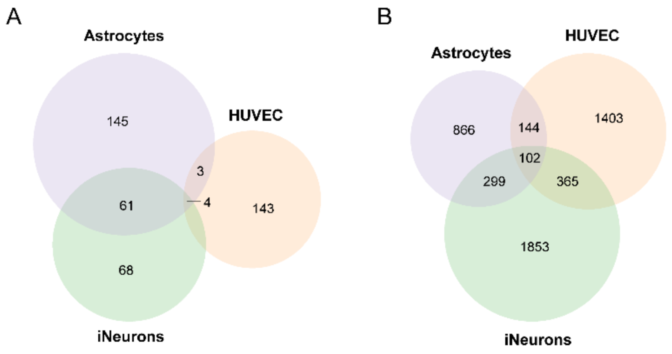

Supplementary figure 4. Venn diagrams showing overlap of A) GO terms and B) differentially expressed (DE) genes identified per cell type (iNeurons, astrocytes, and HUVEC) when comparing 3D to 2D culture systems (adjusted p-value < 0.05).
